# Disparities in Quality of Life by Appalachian-Designation Among Women with Breast Cancer

**DOI:** 10.13023/jah.0102.06

**Published:** 2019-07-06

**Authors:** Robin C. Vanderpool, Ann L. Coker, Heather M. Bush, Sarah E. Cprek

**Keywords:** Appalachia, quality of life, geography, breast cancer, health care, rural, health outcomes, depression, stress, spiritual wellbeing

## Abstract

**Introduction:**

Few studies have examined the association of geography and quality of life (QOL) among breast cancer patients, particularly differences between Appalachian and non-Appalachian Kentucky women, which is important given the cancer and socioeconomic disparities present in Appalachia.

**Purpose:**

The purpose of this study was to determine whether women with breast cancer residing in Appalachian Kentucky experience poorer health outcomes in regards to depression, stress, QOL, and spiritual wellbeing, relative to those living in non-Appalachian Kentucky after adjusting for demographic, socioeconomic, and health-related factors.

**Methods:**

Women, aged 18–79, recruited from the Kentucky Cancer Registry between 2009 and 2013 with an incident, primary breast cancer diagnosis completed a telephone interview within 12 months of diagnosis. In this cross-sectional study, sociodemographic characteristics and mental and physical health status were assessed, including number of comorbid conditions, symptoms of depression and stress, and QOL.

**Results:**

Among 1245 women with breast cancer, 334 lived in Appalachia and 911 in non-Appalachian counties of Kentucky. Appalachian breast cancer patients differed from non-Appalachian patients on race, education, income, health insurance status, rurality, smoking, and stage at diagnosis. In unadjusted analysis, Appalachian residence was associated with having significantly more comorbid conditions, more symptoms of stress in the past month, and lower Functional Assessment of Cancer Therapy-Breast scores compared to non-Appalachian residence.

**Implications:**

However, adjustment for sociodemographic and health-related differences by region appear to explain geographic differences in these poorer QOL indicators for women living in Appalachian Kentucky relative to non-Appalachian Kentucky. Policy-, provider-, and individual-level implications are discussed.

## INTRODUCTION

Kentucky’s national ranking as first in cancer incidence and mortality^1^ is attributed primarily to health and socioeconomic disparities in the 54-county, rural Appalachian region of the state where residents carry a disproportionate burden of many preventable and screenable cancers.^2^,^3^ When examining breast cancer, women in Appalachian Kentucky experience *lower* five-year (2011–2015) incidence rates than their non-Appalachian counterparts (117.2 vs. 128.6) and *elevated* mortality rates (23.9 vs. 20.7).^4^ Further, women in Appalachian Kentucky are diagnosed with breast cancer at later stages (i.e., regional and distant) than women living in non-Appalachia (42.7 vs. 42.5, respectively).^4^ Although regional variations in access to screening and diagnostic services^5^ as well as cultural beliefs (e.g., fatalism) may explain these geographical differences in disease presentation,^6^ this rural region is also impacted by increased socioeconomic deprivation; lower rates of educational achievement; geographic isolation; increased rates of at-risk health behaviors (e.g., smoking) and comorbidities; and limited access to primary care, mental health, and oncology specialists.^2^

These circumstances and environment may lead to increased stress, worry, and decreased quality of life (QOL), particularly following a diagnosis of breast cancer.^7^ When examining breast cancer-related QOL exclusively among rural communities, studies have found patients report high levels of stress and hopelessness, lower QOL and lower functional wellbeing, and increased symptom complaints.^8^,^9^ Although these studies have been useful in determining an association between rurality and QOL among breast cancer patients, none have looked specifically at Appalachian Kentucky. Therefore, the purpose of this study was to determine whether women with breast cancer residing in Appalachian Kentucky experience poorer health outcomes in regards to depression, stress, QOL, and spiritual wellbeing relative to those living in non-Appalachian Kentucky after adjusting for demographic, socioeconomic, and health-related factors.

## METHODS

### Design and Study Sample

Data originated from a larger study focusing on violence against women and its impact on access to care among women diagnosed with cancer in Kentucky.^10^ For this study, the Kentucky Cancer Registry (KCR) was used to identify and recruit women aged 18–79 who had been diagnosed with an incident and primary case of breast, cervical, or colorectal cancer in the previous 12 months. Women were contacted approximately 12 months after their initial diagnosis; the recruitment period extended from November 2009 to December 2013. After confirming a patient’s diagnosis, KCR contacted eligible patients’ physicians to ensure there was no reason the patient should not be approached for study participation. KCR then contacted the women by mail and/or phone in order to determine if they were interested in participating.

The Kentucky Cancer Registry provided information on all women interested in participating to the University of Kentucky (UK) Survey Research Center, who then made contact with the women. Once women were reached via telephone, the interviewer obtained verbal consent before beginning the interview. The average interview duration was 30 minutes; women were offered a $10 incentive.^10^ The study was approved by the UK Institutional Review Board (09-0685-F1V) and a Certificate of Confidentiality was granted (MD-09-007).

### Measures

Varying demographic, socioeconomic, and health-related items were included in the questionnaire to create a profile of female breast cancer patients by region in Kentucky. Stage at diagnosis (Stage 0, 1, 2, 3, 4), age at diagnosis, health insurance status, and county of residence were available from KCR. Each county’s corresponding 2013 Rural–Urban Continuum Code was used to create a rural–urban classification. County of residence was also used to create the dichotomous independent variable of Appalachian or non-Appalachian.

Dependent variables included: (1) comorbid physical conditions at diagnosis; (2) symptoms of depression and stress; and (3) QOL and spiritual wellbeing. Women were asked whether a doctor had ever told them they had additional health conditions (e.g., asthma, high blood pressure, diabetes). Response options for each condition were yes or no. Physical conditions were summed to create an ordinal variable indicating the number of conditions the woman has experienced.

Symptoms of stress were determined using three of the four-item Perceived Stress Scale (PSS). Participants were asked to use this scale to recall perception of stress during the two to three months after initial diagnosis as well as stress the month prior to the interview. Replies were measured on a five-point Likert scale (0=Never…4=Very Often). The Cronbach alpha for the altered PSS measure was 0.63 and 0.60 for the recall periods, respectively.^10^ Depression was measured using five items from the Brief Symptom Inventory (BSI-18) on a five-point scale ranging (0=Not at all to 4=Very Much). Cronbach alpha for the five-item measure was 0.78.^10^

Cancer-related QOL was measured with a 27-question Functional Assessment of Cancer Therapy-Breast Cancer questionnaire (FACT-B; Cronbach’s alpha=0.9).^10^ FACT-B measures physical functioning, social/family functioning, emotional functioning, and functional status as it applies to the past 7 days. Two FACT-B items, which assess the patient’s relationship with her doctor, were excluded from the questionnaire. Response options for the FACT-B were measured on a five-point Likert scale (0=Not at all…4=Very much). Spiritual wellness was determined using the first 12 items from the Functional Assessment of Chronic Illness Therapy–Spiritual Wellbeing Scale (FACIT-Sp) measured on a five-point Likert scale (0=Not at all…4=Very much). The recall period was the last 7 days. The Cronbach’s alpha for FACIT-Sp was 0.85.^10^

### Data Analyses

Sociodemographic and health attributes of breast cancer cases living in Appalachian and non-Appalachian regions were compared to determine covariates for subsequent analyses addressing the associations between geographic region and QOL cancer outcomes using either two-sample t-test for the two continuous measures (age at diagnosis and number of children) or chi-square tests for the remaining characteristics (Table 1). In an effort to parse out the mediational effect of demographic, socioeconomic, and health-related variables on Appalachian residence on QOL cancer outcomes, four sets of models were run: (1) unadjusted; (2) adjusting for age at diagnosis, rurality, race, and stage; (3) additionally adjusting for income and private health insurance; and (4) additionally adjusting for current smoking and education.

ANOVA analyses were used to compare outcomes for Appalachian (exposed) versus non-Appalachian (non-exposed) residence; adjustments for covariates were made using ANCOVA. These analyses were performed separately for the dependent variables of number of covariates, total FACT-B score, and FACIT-Sp. A similar analysis was performed using MANOVA without adjustments and MANCOVA with adjustments for dependent variables for stress and depression as well as for the domains of the FACT-B score because these outcomes were correlated. For models using each outcome variable, the t-statistic, df, and p-value for the effect of Appalachian residence is provided. Analyses were completed in 2016 using in SAS® Version 9.3 (Cary NC); p-values ≤ 0.05 were considered statistically significant.

## RESULTS

Of the 4628 women diagnosed with breast cancer and included in the KCR between November 2009 and December 2013, we could not survey 1414 women (30.6%) because 42 had died, physicians requested that 24 cases not be contacted, and 1348 could not contacted by phone or mail. Another 1969 women (42.5%) refused participation. Response rates (n=1245) were 26.9% of all 4628 women diagnosed with breast cancer, or 38% of 3280 we contacted for active consent. Response rates were higher among Appalachian Kentucky women (29.9%) than non-Appalachia women (25.9%) (χ2=6.85; p=0.01 for 2-tail test).

Women with breast cancer who lived in Appalachian Kentucky differed from those living in non-Appalachia on race, education, income, health insurance status, rurality, smoking, and stage at diagnosis (Table 1). Among Appalachian Kentucky women, there was less racial diversity, lower rates of college-level education and upper monthly income, higher rates of being uninsured or covered by government-sponsored insurance, a higher likelihood of living in a very rural county, higher rates of current smoking, and increased rates of Stage 4 disease. While no regional differences in the age of diagnosis were identified, age was included as a covariate in subsequent models because age was associated with several of the QOL outcomes.

Appalachian women reported more physical comorbidities, higher stress levels at diagnosis and within the past month, and a lower FACT-B total score (and lower individual domain scores) indicating decreased QOL as compared to non-Appalachian patients (Table 2). Further analyses used Wilks’ Lambda to indicate the appropriateness of MANOVA for correlated outcomes if the associated is p <0.05. For the MANOVA model including depression and stress at two time frames, the Wilks’ Lambda was nonsignificant (F =2.32 _df 3,1237_^p=0.08^) and the Wilks’ for MANOVA for the four FACT-B subscales was significant (F=3.69 _4,1220_^0.005^). In the unadjusted model (model ^a^), Appalachian residence was associated with more comorbid physical conditions, more symptoms of stress in the past month, and poorer cancer-related QOL as measured with the total FACT-B score and all but the social domain for FACT-B subscales. No regional differences in symptoms of depression or stress at diagnosis were observed; similarly, FACIT-Sp scores did not differ by region. These patterns generally held when adjusting for age at diagnosis, rurality, race, and stage (model ^b^), yet were not significant when additionally adjusting for income and private insurance (model^c^). The addition of smoking and education to the final model (model ^d^) suggests that these two covariates did not explain patterns beyond adding income and insurance in the prior models (model ^b and c^).

## IMPLICATIONS

To our knowledge, this is one of the first studies to specifically explore cancer-related QOL differences between women with breast cancer residing in Appalachian versus non-Appalachian Kentucky. We found that Appalachian women were more likely to live in extremely rural communities, be of lower socioeconomic status (SES), and experience poor health outcomes such as higher rates of smoking, Stage 4 disease, physical comorbidities, and stress compared to their non-Appalachian counterparts. In reviewing the unadjusted mean scores, Appalachian women also had lower FACT-B total scores (2.55-point difference). However, after adjustment for sociodemographic and cancer attributes, women living in Appalachian Kentucky did not have poorer cancer-related QOL compared to women residing in non-Appalachia. Adjustment for age at diagnosis, rurality, race, stage, income, and insurance status appear to mediate or explain regional differences in cancer-related QOL noted in the unadjusted comparisons. Specifically, income and private insurance are likely the important mediators explaining Appalachian regional differences in cancer-related QOL because their addition to models resulted in no observed regional differences in the noted outcomes. These findings support Schootman et al. who found geographic differences in rates of depression and social support were not significant once SES, access to medical care, or other chronic conditions were included in the analysis.^11^

Overall, the findings reiterate the powerful influence of SES on breast cancer outcomes, including QOL^7^,^12^ and the need to focus on improving education, income, health insurance coverage, and employment opportunities as well as access to physical and mental health services. In parallel with these policy-related implications, there are also individual- and provider-level considerations. There were differences in the prevalence of smoking and later stage breast cancer diagnoses among Appalachian women indicating the need for evidence-based, culturally appropriate tobacco prevention/cessation and mammography services in the region. Additionally, the difference in unadjusted associations between geographic region and QOL measures has relevance for those formally and informally caring for cancer patients. Appalachian breast cancer patients may present with more comorbid conditions, increased acute and chronic stress, and limited physical functioning across treatment and recovery. Clinical and social support networks that address differences in mental and physical health trajectories may reduce regional differences in cancer-related QOL.

Although this study is a unique contribution to the breast cancer QOL literature, particularly its focus on Appalachia, there are noted limitations in the cross-sectional methodology. A primary limitation is collecting several of the sociodemographic variables and defining QOL based on women’s self-report, which may be biased; yet women are the ultimate authority on their own QOL and mental health. Those completing interviews (38% of women we were able to contact) may differ from those who did not participate on attributes we could and could not measure. For example, KCR did not provide specific data on stage or age for those women who did not complete the survey. We were able to document that Appalachian women were more likely to agree to be interviewed than those living in non-Appalachia; however, this modest difference is unlikely to bias the consistently null findings observed here. Literature comparisons were generated from U.S. rural versus urban cancer QOL studies, which may not translate directly to Appalachian and non-Appalachian areas of Kentucky. Study limitations are countered with strengths, including use of the same interview protocol for all participants and use of outcome measures with strong psychometric properties, thereby limiting measurement bias. Sampling from KCR improved study power and sample representativeness. Moreover, the study provides a foundation for future research examining psychological and other predictors of breast cancer-related QOL outcomes in Kentucky as well as the entire 13- state Appalachian region, including assessments of rural and urban counties, Appalachian subregions, and non-Appalachian areas.

**SUMMARY BOX**

**What is already known about this topic?**

Rural-residing breast cancer patients have previously reported higher levels of stress and hopelessness, lower quality of life (QOL) and lower functional wellbeing, and increased symptom complaints.

**What is added by this report?**

Few studies have specifically examined differences in QOL between Appalachian and non-Appalachian Kentucky women diagnosed with breast cancer. Adjustment for sociodemographic and health-related outcomes by geographic region appear to explain differences in poorer QOL indicators for women in Appalachian Kentucky relative to non-Appalachian Kentucky.

**What are the implications for public health practice, policy, and research?**

Socioeconomic status (SES) is a powerful influence on breast cancer outcomes, including QOL. Additional research is needed to understand the complex interplay between SES, geographic residence, mental health status, and cancer.

**Table 1:** Participant characteristics (N = 1245)

	Appalachian (n = 334)	Non-Appalachian (n = 911)	t-test _df_^p value^
**Mean age at diagnosis (SE)**	57.33 (0.54)	56.34 (0.33)	1.55 _1244_^NS^
**Mean number of children (SE)**	2.13 (0.07)	2.09 (0.04)	0.46 _1243_^NS^
**Percent non-white**	4.2%	8.1%	5.75 _1_^0.01^
**Percent currently married**	69.5%	67.7%	0.35 _1_^NS^
**Education**			54.84 _4_^<0.0001^
Less than high school graduate	15.6%	5.4%	
High school graduate-GED	36.8%	30.8%	
Some college	15.3%	19.0%	
Vocational school or Assoc Degree	14.7%	12.6%	
College graduate or more	17.7%	32.2%	
**Monthly Household Income**			42.05 _5_^<0.0001^
Less than $1,000	13.8%	8.2%	
$1,000–$1,999	30.8%	18.9%	
$2,000–$2,999	17.1%	15.5%	
$3,000–$3,999	10.5%	14.3%	
$4,000–$4,999	11.4%	14.8%	
$5,000 or more	16.5%	28.2%	
**Health Insurance**			32.83 _3_^<0.0001^
No insurance of any kind	5.1%	1.7%	
Medicaid vs. no Medicaid	10.2%	6.8%	
Medicare vs. no Medicare	32.6%	23.4%	
Private vs. other or no coverage	52.1%	68.2%	
**2013 Rural-Urban Continuum Coding**			439.91 _4_^<0.0001^
Metro (≥1 million) [Code = 1]	0.0%	51.2%	
Metro (<250,000–1 million) [Code = 2]	14.1%	25.5%	
Urban (pop 20,000–250,000) [Codes = 3–5]	14.7%	5.6%	
Urban (pop 2,500–19,999) [Codes = 6–7]	49.7%	14.7%	
Rural (<2500) [Codes = 8–9]	22.3%	3.0%	
**Smoking Status**			9.00 _2_^0.01^
Current smoker	16.8%	10.7%	
Former smoker	27.8%	32.1%	
Never smoker	55.4%	57.3%	
**Stage at Diagnosis**			14.08 _4_^0.007^
In situ (0)	2.1	5.3	
Stage 1	68.0	65.8	
Stage 2	1.2	1.5	
Stage 3	24.0	25.6	
Stage 4	4.8	1.9	

**Table 2:** Cancer-related quality of life measures: unadjusted mean score (standard error) by Appalachian or non-Appalachian Kentucky region and Appalachian residence unadjusted and adjusted models

	Unadjusted Mean Score (SE)		t test _df_^p value^ for Appalachian Residence Model			
	Kentucky County of Residence					
	Appalachian (n=334)	Non-Appalachian (n=911)	Unadjusted^a^	Adjusted b	Adjusted c	Adjusted d
**Number of comorbid physical conditions at diagnosis**	1.82 (0.07)	1.55 (0.04)	3.45 _1239._^0006^	1.97 _1239_^.05^	1.39 _1238_^NS^	1.25 _1238_^NS^
**Symptoms of Depression and Stress Score**						
Depression at diagnosis	1.65 (0.09)	1.65 (0.06)	−0.07 _1240_^NS^	0.26 _1240_^NS^	−0.37 _1239_^NS^	−0.58 _1239_^NS^
Stress at diagnosis	4.50 (0.17)	4.48 (0.10)	0.13 _1240_^NS^	−0.41 _1240_^NS^	−0.81 _1239_^NS^	−0.92 _1239_^NS^
Stress in the past month	3.46 (0.14)	3.09 (0.09)	2.18 _1240_^.03^	2.18_1240_^.03^	1.47 _1239_^NS^	1.36 _1239_^NS^
**Functional Assessment of Cancer Therapy-Breast Cancer (FACT-B)**						
FACT-B Total Score	63.94 (0.69)	66.49 (0.42)	−3.30 _1223_^.003^	−2.50 _1223_^.01^	−1.56 _1223_^NS^	−1.35 _1223_^NS^
FACT physical domain	14.45 (0.26)	15.63 (0.16)	−3.80 _1224_^.0001^	−2.41 _1224_^.02^	−1.57 _1223_^NS^	−1.42 _1223_^NS^
FACT social domain	18.38 (0.18)	18.64 (0.11)	−1.24 _1224_^NS^	−1.08 _1224_^NS^	−0.40 _1223_^NS^	−0.28 _1223_^NS^
FACT emotional domain	13.81 (0.19)	14.29 (0.12)	−2.18 _1224_^.03^	−1.70 _1224_^NS^	−1.06 _1223_^NS^	−0.85 _1223_^NS^
FACT functional domain	17.31 (0.22)	17.85 (0.13)	−2.09 _1224_^.04^	−2.61 _1224_^.01^	−1.71 _1223_^NS^	−1.50 _1223_^NS^
Functional Assessment of Chronic Illness Therapy-Spiritual Well-being Scale (FACIT-Sp)						
FACIT – Sp Score	31.28 (0.28)	31.76 (0.17)	−1.51 _1224_^NS^	−1.93 _1224_^.05^	−1.26 _1223_^NS^	−1.01 _1223_^NS^

a Unadjusted. MANOVA Wilks’ Lambda F-statistic (df1, df2)^p-value^ for depression/stress: 2.30 _3,1237_^.08^; MANOVA Wilks’ Lambda F-statistic (df1, df2)^p-value^ for FACT: 3.69 _4,1220_^.005^

b Adjusting for age at diagnosis, rurality, non-white race, and stage. MANOVA Wilks’ Lambda F-statistic (df1, df2)^p-value^ for depression/stress: 2.52 _3,1232_^.06^; MANOVA Wilks’ Lambda F-statistic (df1, df2)^p-value^ for FACT: 1.96 _4,1216_^NS^

c Additionally adjusting for income and private health insurance. MANOVA Wilks’ Lambda F-statistic (df1, df2)^p-value^ for depression/stress: 1.79 _3,1229_^NS^; MANOVA Wilks’ Lambda F-statistic (df1, df2)^p-value^ for FACT: 0.93 _4,1213_^NS^

d Additionally adjusting for above and current smoking and education. MANOVA Wilks’ Lambda F-statistic (df1, df2)^p-value^ for depression/stress: 1.81 _3,1227_^NS^; MANOVA Wilks’ Lambda F-statistic (df1, df2)^p-value^ for FACT: 0.75 _4,1211_^NS^
